# Identification of candidate genes associated with porcine meat color traits by genome-wide transcriptome analysis

**DOI:** 10.1038/srep35224

**Published:** 2016-10-17

**Authors:** Bojiang Li, Chao Dong, Pinghua Li, Zhuqing Ren, Han Wang, Fengxiang Yu, Caibo Ning, Kaiqing Liu, Wei Wei, Ruihua Huang, Jie Chen, Wangjun Wu, Honglin Liu

**Affiliations:** 1 Department of Animal Genetics, Breeding and Reproduction, College of Animal Science and Technology, Nanjing Agricultural University, Nanjing, 210095, China; 2 Key Laboratory of Swine Genetics and Breeding, Ministry of Agriculture, Key Lab of Agriculture Animal Genetics, Breeding and Reproduction, Ministry of Education, College of Animal Science, Huazhong Agricultural University, Wuhan, Hubei, 430070, China; 3 The Cooperative Innovation Center for Sustainable Pig Production, Huazhong Agricultural University, Wuhan, Hubei, 430070, China

## Abstract

Meat color is considered to be the most important indicator of meat quality, however, the molecular mechanisms underlying traits related to meat color remain mostly unknown. In this study, to elucidate the molecular basis of meat color, we constructed six cDNA libraries from *biceps femoris* (Bf) and *soleus* (Sol), which exhibit obvious differences in meat color, and analyzed the whole-transcriptome differences between Bf (white muscle) and Sol (red muscle) using high-throughput sequencing technology. Using DEseq2 method, we identified 138 differentially expressed genes (DEGs) between Bf and Sol. Using DEGseq method, we identified 770, 810, and 476 DEGs in comparisons between Bf and Sol in three separate animals. Of these DEGs, 52 were overlapping DEGs. Using these data, we determined the enriched GO terms, metabolic pathways and candidate genes associated with meat color traits. Additionally, we mapped 114 non-redundant DEGs to the meat color QTLs via a comparative analysis with the porcine quantitative trait loci (QTL) database. Overall, our data serve as a valuable resource for identifying genes whose functions are critical for meat color traits and can accelerate studies of the molecular mechanisms of meat color formation.

Although meat quality is difficult to define accurately, it can be evaluated by multiple technology indicators, including color, pH, water-holding capacity (WHC), drip loss, tenderness, intramuscular fat content, glycolytic potential (GP) as well as by flavor, microbial spoilage, and contamination^1^. Among these attributes, meat color has been identified as the most important appearance indicator of meat quality. Meat color provides consumers with their first visual impression of the product and directly influences their purchasing decisions^2^. Although the economic impacts of discoloration-induced meat wastage on agriculture remains to be objectively assessed, there is no doubt that discoloration of fresh meat results in financial losses. As living standards improve, the demand for high-quality pork is increasing in most countries. Accordingly, meat color traits should be considered as a breeding target by breeders.

Meat color is a complex quantitative trait which is influenced by numerous genes and environmental factors. Previous research showed that meat color trait is characterized by low heritability (0.14–0.25)^3^, and it is hard to accurately measure in live animals. Consequently, it is difficult to achieve genetic improvement in this trait by traditional breeding methods. However, recent progress in genomics has led to the identification of more and more chromosomal regions, genes, and mutations underlying phenotypic variation of domestic animals. These advances drive the application of marker-assisted selection (MAS) in the field of animal breeding.

To date, 15,108 pig quantitative trait loci (QTLs) representing 600 different traits have been documented in the Pig QTL Database. Among these QTLs, 571 are associated with meat color (www.animalgenome.org, release 29, May 2016). Initially, most meat color QTLs covering relatively large genomic regions were identified using linkage mapping methods^4^,^5^,^6^,^7^, and the fine mapping work for meat color QTLs were subsequently conducted^8^,^9^. Recently, with the development of high-density SNP chips and genome sequencing, genome-wide association studies (GWAS) have provided a more precise method for identifying the genomic regions and markers associated with meat color^10^,^11^,^12^,^13^,^14^. Previously, a handful of causative genes and quantitative trait nucleotides (QTN) were demonstrated to influence meat color. These include *PRKAG3*, a major gene responsible for acid meat^15^,^16^,^17^, and *RYR1*, a major gene responsible for Pale, Soft, Exudative (PSE) meat^18^,^19^. In addition, some novel genes, such as *NUDT7*^8^, *EDN3*, and *PHACTR3*^20^, were also associated with meat color. Despite this progress, our knowledge of the genetic factors underlying variation in meat color remains incomplete.

In this study, to identify the candidate genes that influence meat color traits, we performed a comparative analysis of the whole transcriptomes of *biceps femoris* (Bf; white muscle) and *soleus* (Sol; red muscle), which are characterized by obvious color differences, using RNA-seq technology. We identified a series of differentially expressed genes (DEGs) between these two tissue types, which represent potential candidate genes affecting meat color traits. Using these data, we identified several important GO terms and metabolic pathways associated with meat color. Overall, our data provide a solid foundation for identifying the critical genes whose functions affect meat color traits, and can facilitate studies of the molecular regulatory mechanisms underlying meat color.

## Results and Discussion

### Phenotypic confirmation

**Figure 1:**
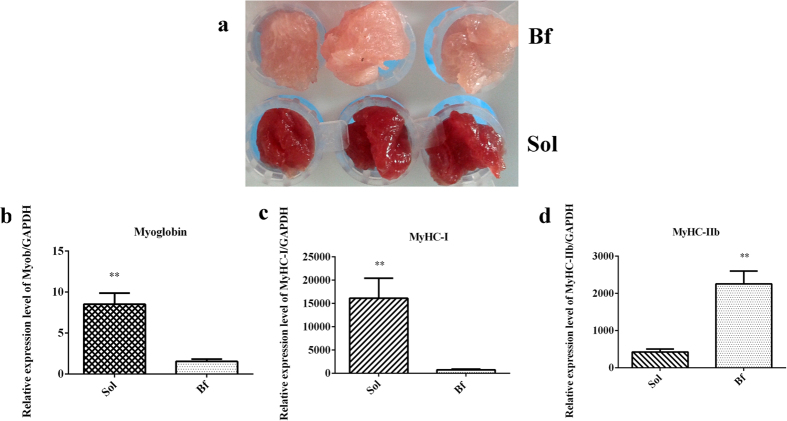
Phenotypic characterization. **(a)** Meat color of *Biceps femoris* (Bf) and *Soleus* (Sol). **(b**–**d)** Relative expression levels of *myoglobin*, *MyHC-I*, and *MyHC-IIb* genes between Bf and Sol muscles, respectively. Real-time PCR is used to detect the expression level, and the gene expression levels are normalized by reference gene *GAPDH*. The unpaired Student’s *t*-test is used to evaluate the statistical significance of differences, *P ≤ 0.05, **P ≤ 0.01. All data are presented as mean ± standard error (SE).

To avoid individual differences between animals to the greatest extent possible, the subjects for this study were three full-sib female pigs with similar performances (Supplementary Table S1). The source of tissues were derived from Bf and Sol in this study. Bf (light or pale) is a typical fast-twitch fiber characterized by more higher glycolytic potential, and Sol (dark or red) is a typical slow-twitch fiber characterized by more higher oxidative potential^21^. The differences in meat color between Bf and Sol muscles were obvious, and were further confirmed by quantitation of *myoglobin*, *MyHC-I*, and *MyHC-IIb* expression (Fig. 1a). The results revealed that these genes were expressed at significantly different levels in Bf and Sol muscles (*P* < 0.01) (Fig. 1b–d). Taken together, these results suggest that Bf and Sol muscles are suited for candidate gene screening of meat color.

### Overall statistics of sequencing data

We constructed six cDNA libraries (three from Bf muscles: Bf28, Bf35, and Bf36, and three from Sol muscles: Sol28, Sol35, and Sol36) and sequenced them on a HiSeq 4000 platform. A summary of sequencing data indicated that each library had highly consistent statistical parameters (Table 1). The GC content of each library was more than 54%, and the ratio of Q20 bases (those with a base quality >20 and error rate <0.01) was higher than 95%, indicating that high- quality raw reads were obtained from each library. After strict filtering, more than 6 Gb clean bases were obtained for each library, and the clean base ratio of each library was approximately 99.6%. When the data were mapped onto the reference genome, the mapped reads ratio for each library was greater than 72%.

### Identification of novel transcripts and refinement of gene structures

In total, 311 novel transcripts were predicted from six cDNA sequencing libraries (Supplementary Table S3), significantly fewer than the number of novel transcripts predicted from porcine muscle cDNA libraries by Zhu *et al*.^22^. These differences may be due to differences in software used in both studies, or the relatively stringent criteria used in this study. Protein-coding and non-coding transcripts were distinguished with CNCI software. Among the novel transcripts, 61 were protein-coding, 250 were non-coding; and the coding transcripts had a higher CNCI score than the non-coding transcripts, which is consistent with the results obtained from a previous study^22^. To improve gene annotation information in the current database, the 5′ and 3′ boundaries of known genes were refined by alignment of known transcripts with reconstructed transcripts from transcriptome sequencing data. In total, 1707 genes were thus refined, including 1252 genes refined at the 5′ region, 1195 genes refined at the 3′ region, and 740 genes refined at both end regions. Detailed annotation information regarding structurally refined genes is provided in Supplementary Table S4.

### Alternative splicing analysis

**Figure 2:**
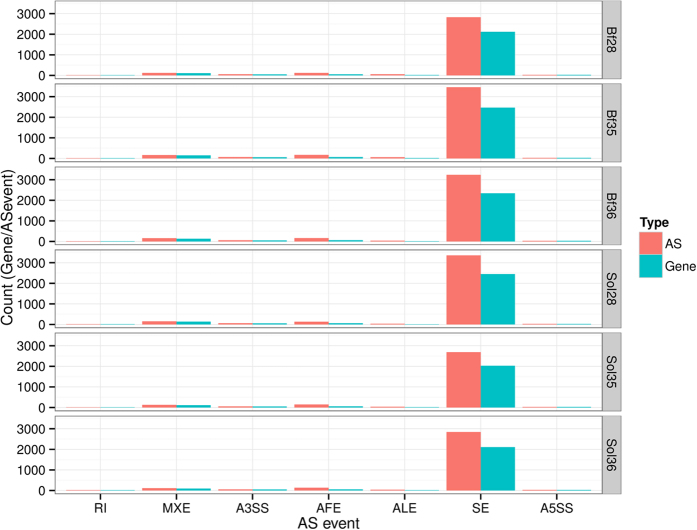
Statistics of alternative splicing events. X axis indicates the types of AS, SE: skipping exon; AFE: alternative first exon; A3SS: alternative 3′ splice site; MXE: mutually exclusive exon; ALE: alternative last exon; RI: intron retention; A5SS: alternative 5′ splice site. Y axis indicates the gene or AS numbers. Each row indicates one sample.

Alternative splicing (AS) generates multiple transcripts from the same gene, some of which may perform different functions. We used the rMATS software to analyze the AS events in each library^23^. Seven types of AS events were detected: skipping exon (SE), alternative first exon (AFE), alternative 3′ splice site (A3SS), mutually exclusive exon (MXE), alternative last exon (ALE), intron retention (RI), and alternative 5′ splice site (A5SS). The most common of these was SE event, and consistent with the results of previous studies, although the ratio of SE to all AS events was very different from that obtained by Zhu *et al*.^22^. In the present study, SE accounted for more than 85% of all AS events in each library (Fig. 2 and Supplementary Table S5).

### Gene expression analysis

Expression levels of all genes were calculated using the HTseq software, and described by the reads per kilobase per million reads (RPKM). In total, of 18,959 genes were detected in six cDNA libraries, and the number of expressed genes in each library was similar among libraries (16,241–16,841) (Supplementary Table S6). In this study, 83, 86, 92, 89, 79, and 86 highly expressed genes with RPKM >1000 were determined in the Bf28, Bf35, Bf36, Sol28, Sol35 and Sol36 libraries, respectively. These genes are mainly involved in skeletal muscle structure and contraction, mitochondrial function, energy metabolism, and ribosomal function. *ACTA1* (ENSSSCG00000010190), the most highly expressed gene in each library, encodes an essential component of skeletal muscle cell structures (sarcomeres) that plays an important role in muscle contraction. Ten of these highly expressed genes were also DEGs (see next section): *MYH7* (ENSSSCG00000002029), *TPM3* (ENSSSCG00000006556), *MLC2V* (ENSSSCG00000009830), *ACTN2* (ENSSSCG00000010144), *MYL3* (ENSSSCG00000011325), *TNNC1* (ENSSSCG00000011441), *FHL1C* (ENSSSCG00000012699), *LMOD2* (ENSSSCG00000016605), *TNNT1* (ENSSSCG00000024061), *TNNI1* (ENSSSCG00000025353). The functional roles of these genes in meat color trait remain to be elucidated.

### Validation of DEGs and cluster analysis

**Figure 3:**
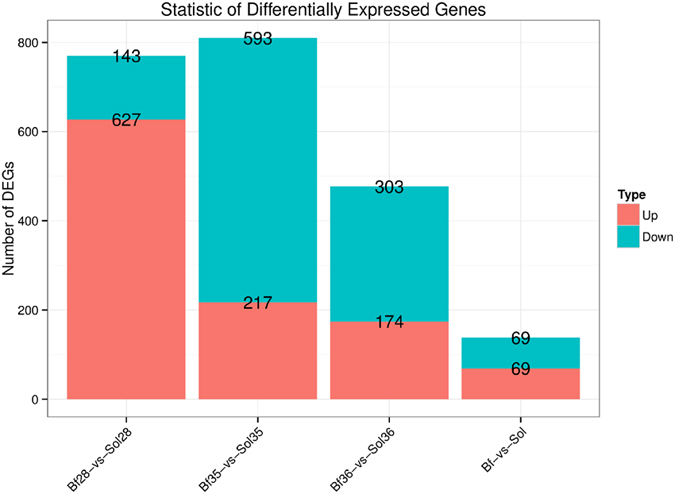
Statistics of DEGs from DEseq2 and DEGseq methods. Bf-vs-Sol indicates the comparative strategy by DEseq2 method in X axis; Bf28-vs-Sol28, Bf35-vs-Sol35 and Bf36-vs-Sol36 indicate the comparative strategy by DEGseq method in X axis. Y axis indicates the gene number. Red and blue color represent significantly up-regulated and down-regulated genes, respectively.

**Figure 4:**
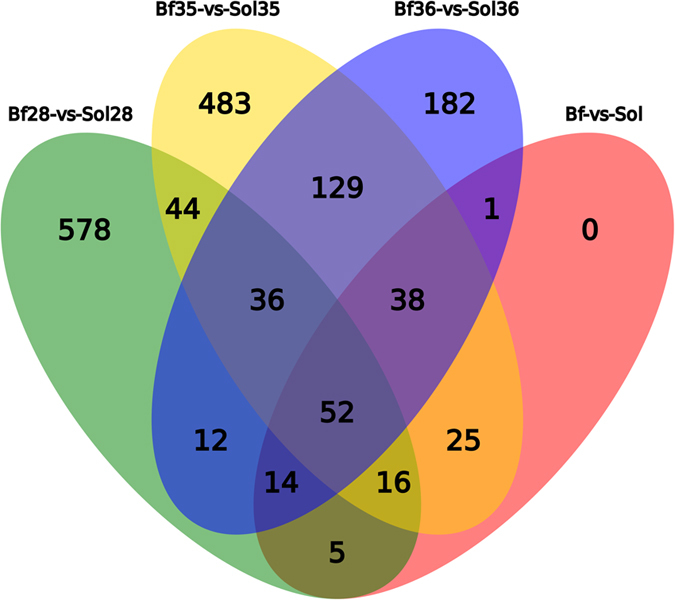
Venn diagram of DEGs between Bf and Sol muscles. A total of 52 overlapped DEGs are obtained by DEseq2 and DEGseq methods. Different color represents different combination, the number in the overlap region represents the overlapped DEGs number.

To screen for critical candidate genes related to meat color traits, we identified DEGs between Bf and Sol muscles using two methods, DEseq2 and DEGseq. DEseq2, which is suitable for analyzing data from biological repeated experiments, identified 138 DEGs in this study (Fig. 3 and Supplementary Table S7). In this study, the samples Bf28 and Sol28, Bf35 and Sol35, and Bf36 and Sol36 represent different skeletal muscles of three different individuals (animals 28, 35, and 36, respectively). To eliminate differences due to genetic background, we used the DEGseq method, which is suitable for analyzing data from the experiments with no biological repeats. In particular, DEGseq was used for the following comparisons: Bf28 vs Sol28, Bf35 vs Sol35, and Bf36 vs Sol36, yielding 770, 810 and 477 DEGs, respectively (Fig. 3 and Supplementary Table S8). Furthermore, 52 overlapped DEGs were filtered out from the DEGs identified by both methods (Fig. 4 and Table 2).

**Figure 5:**
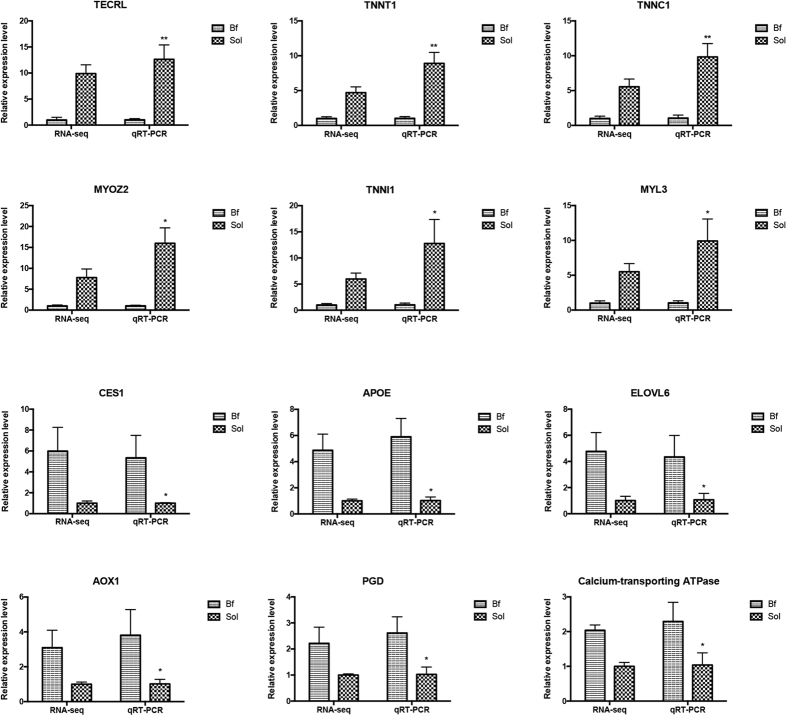
Validation of DEGs by real-time PCR. Bf: *Biceps femoris*; Sol: *Soleus*. RPKM values are used to calculate the gene expression in RNA-seq and normalize the expression of one group to “1”. In real-time PCR, relative expression levels are calculated using ∆∆Ct value method and normalized by reference gene *GAPDH*, and similarly normalize the expression of one group to “1”. The data showed in Y axis represented the fold change. The unpaired Student’s *t*-test is used to evaluate the statistical significance of differences between the two groups, *P ≤ 0.05, **P ≤ 0.01. All data are presented as mean ± standard error (SE).

To experimentally validate the DEGs identified from the sequencing data, we analyzed 12 DEGs (six up-regulated and six down-regulated) using real-time PCR. The results confirmed that the expression patterns of these DEGs were consistent with those obtained from transcriptome sequencing data (Fig. 5). Furthermore, the fold_change values from the two methods were highly correlated (Pearson correlation coefficient R = 0.97) at a high level of statistical significance (*P* < 0.01). These results indicate that the DEGs identified in the genome-wide transcriptome sequencing data are reliable.

**Figure 6:**
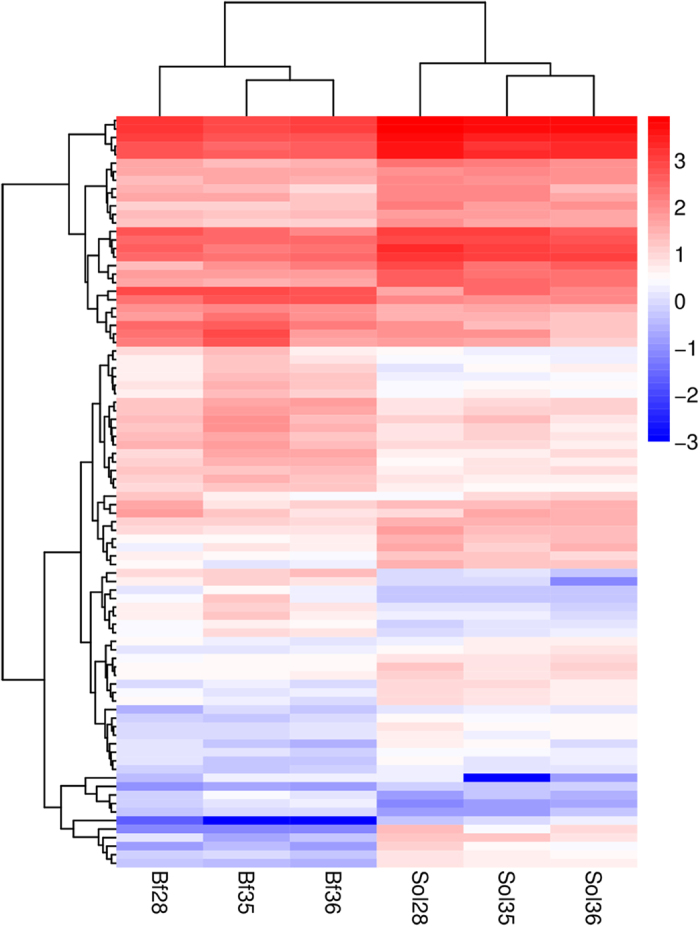
Heatmap analysis of DEGs between Bf and Sol muscles. Heatmap analysis were conducted with 88 overlapped DEGs among three different comparative groups (Bf28-vs-Sol28, Bf35-vs-Sol35 and Bf36-vs-Sol36). Each column represents a sample, and each row represents a DEG. Red and blue gradient indicate an increase or decrease in gene expression abundance, respectively. The corresponding DEGs are showed in Supplementary Table S9.

To obtain insight into the expression patterns of DEGs in the six libraries, we performed a hierarchical cluster analysis based on expression abundance using the Pheatmap software in R package (Supplementary Table S9). As shown in Fig. 6, Bf28, Bf35, and Bf36 were clustered into a group, in which the DEGs exhibited similar expression patterns, and Sol28, Sol35, and Sol36 clustered into another group. Thus the cluster analysis indicated a significant difference in gene expression between Bf and Sol tissue.

### GO and KEGG pathway enrichment analysis of DEGs

**Figure 7:**
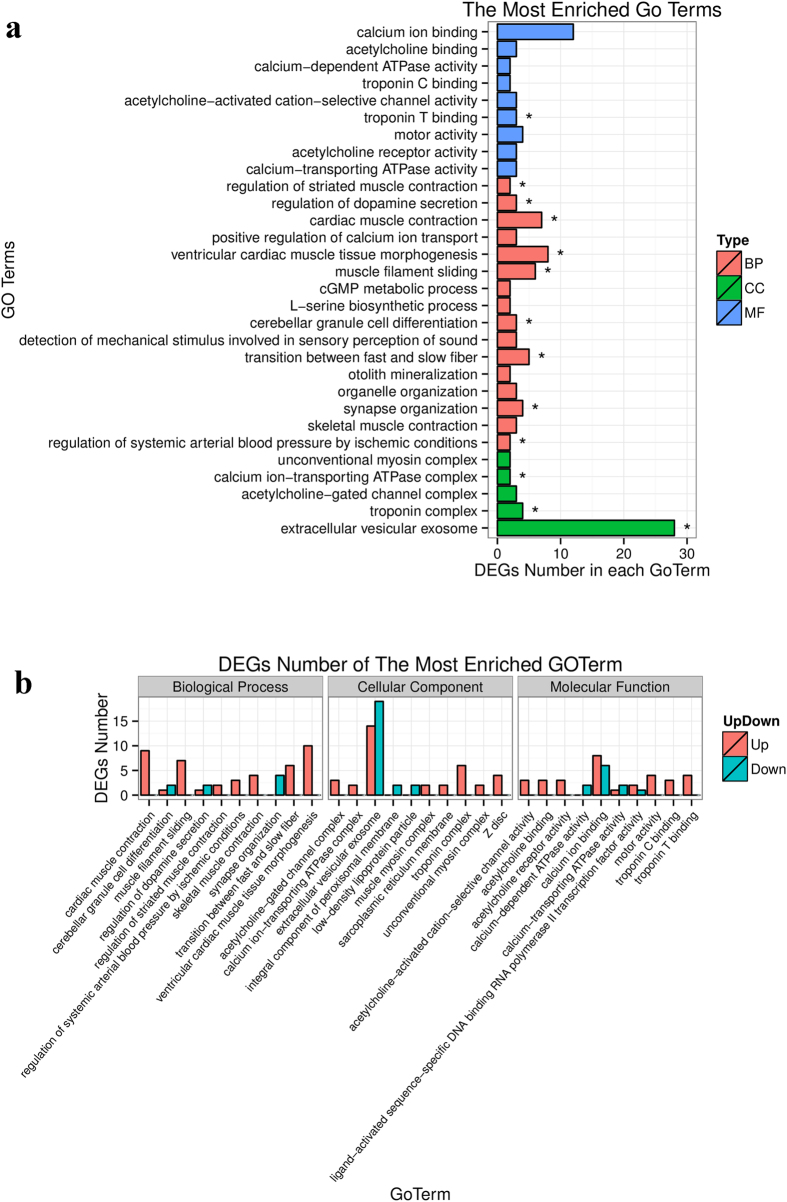
Enriched GO terms and corresponding DEGs numbers of each term for Bf-vs-Sol. **(a)** The most enriched top 30 Go terms, BP: biological process; CC: cellular component; MF: molecular function. **(b)** DEGs number of the most enriched 10 GO terms derived from BP, CC and MF, respectively.

The DEGs identified above represent candidate genes critical for the formation of meat color. To further elucidate the functional roles of DEGs in meat color, we performed GO enrichment analysis of DEGs obtained by the DEseq2 and DEGseq methods using the GOseq software. In total, 13 GO terms were significantly enriched in DEGs obtained by the DEseq2 method (*P* < 0.05), including three terms in cellular component (CC), nine terms in biological process (BP) and one term in molecular function (MP) (Fig. 7). The most abundant term was “extracellular vesicular exosome,” containing 28 DEGs. Other enriched terms, including “troponin complex,” “calcium ion-transporting ATPase complex,” “transition between fast and slow fiber,” “regulation of systemic arterial blood pressure by ischemic conditions,” “regulation of striated muscle contraction,” and “troponin T binding,” refer to properties and pathways potentially associated with meat color (Supplementary Table S10). The most notable term was related to “transition between fast and slow fiber”. Myoglobin is the sarcoplasmic heme protein primarily responsible for the meat color, and the content of myoglobin is higher in type I muscle fiber than in type II b muscle fiber. Indeed, we validated this phenotypic variation in this study (Fig. 1). Accordingly, the difference between muscle fiber types is considered to be a critical factor determining meat color^1^. The Bf and Sol muscles, which are characterized by remarkable differences in contractile and metabolic properties, comprise different muscle fiber types. Several terms related to the contractile and metabolic properties of muscles were also enriched, including “calcium ion-transporting ATPase complex” and “regulation of striated muscle contraction”.

We also performed GO enrichment analysis of DEGs from the DEGseq comparisons Bf28 vs Sol28, Bf35 vs Sol35, and Bf36 vs Sol36. Notably, the term “transition between fast and slow fiber” was significantly enriched in all three comparisons (Supplementary Fig. S2, Supplementary Fig. S3 and Supplementary Fig. S4). The four redox states of myoglobin, namely deoxymyoglobin (DeoxyMb), oxymyoglobin (OxyMb), carboxymyoglobin (COMb), and metmyoglobin (MetMb), are critical for meat color, and the generation of these four compounds is closely related to the significantly enriched term “oxidation-reduction process,” (Supplementary Fig. S3 and Supplementary Fig. S4). Additionally, the states of myoglobin are largely determined by the concentration of O_2_, which is controlled via oxygen consumption^24^; O_2_ metabolism mainly occurs within the mitochondria. Consistent with this, the term “mitochondrion,” containing 48 DEGs, was significantly enriched in the present study (Supplementary Fig. S3). The detailed results of GO enrichment analysis of DEGs from the Bf28 vs Sol28, Bf35 vs Sol35, and Bf36 vs Sol36 comparisons are provided in Supplementary Table S11.

**Figure 8:**
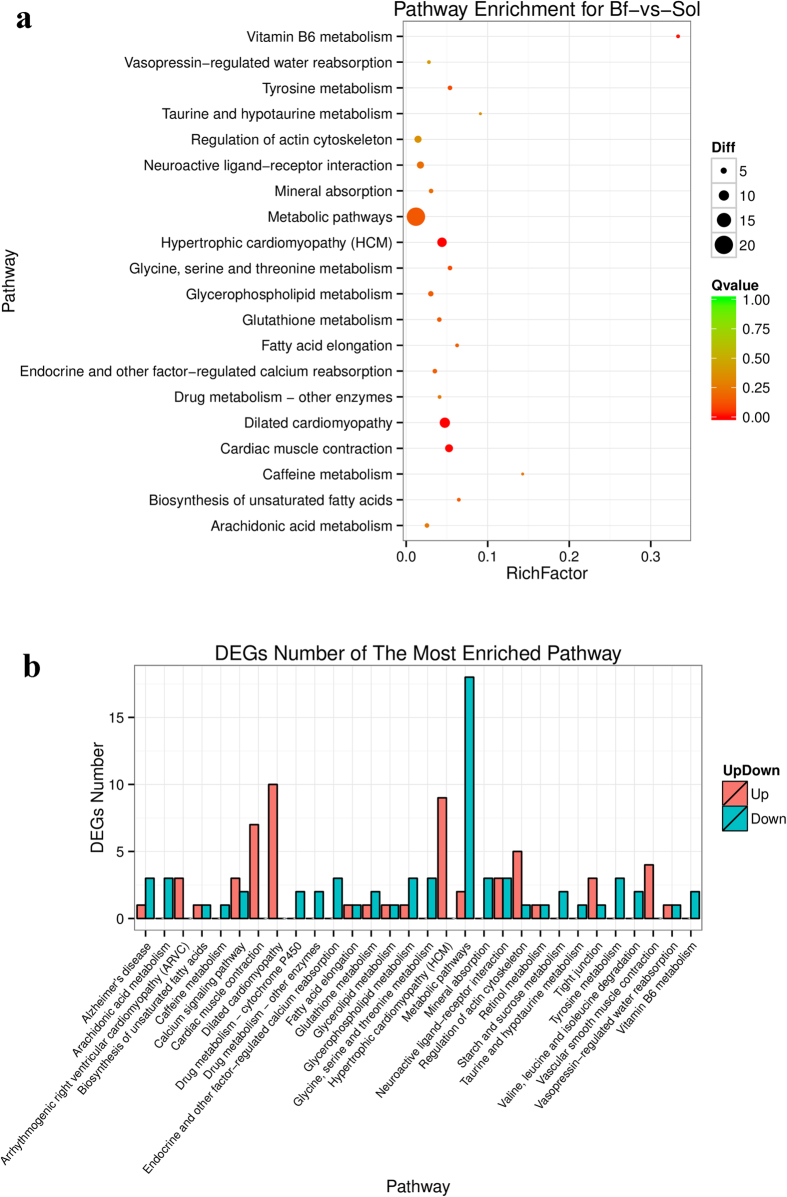
Enriched pathways and corresponding DEGs number of each pathway for Bf-vs-Sol. **(a)** The most enriched 20 pathways, the size of black circle represents the DEGs number; Different color represents the significant test Q value. **(b)** DEGs number of the most enriched 30 pathways.

Meat color is a complex trait; its formation may involve coreaction among many intracellular signaling pathways. To better understand the biological functions and interaction of genes, we conducted KEGG pathway enrichment analysis of DEGs identified by the DEseq2 and DEGseq methods. In total, 115 DEGs obtained from the DEseq2 method were mapped to 95 KEGG pathways, and 12 of these KEGG pathways were significantly enriched (*q* ≤ 0.1); “metabolic pathway (ko01100)” contained the most DEGs (20 unigenes) (Supplementary Table S12 and Fig. 8). Of these pathways, “vitamin B6 metabolism,” “glutathione metabolism,” “biosynthesis of unsaturated fatty acids,” and “fatty acid elongation” could all be related to the formation of meat color. The “vitamin B6” pathway (ko00750) plays an important role in amino acid, glucose, lipid metabolism, and hemoglobin synthesis^25^, and thus may induce several endogenous factors contributing to meat color, such as pH, and lipid oxidation^24^. Antioxidants and the reactive products of lipid oxidation are also known to influence meat color stability^26^. Glutathione is an important antioxidant in animals, and the “glutathione metabolism” pathway was also enriched in the present study. Moreover, “biosynthesis of unsaturated fatty acids” and “fatty acid elongation,” which are involved in lipid oxidation, also influence meat color.

KEGG pathway enrichment analysis of DEGs obtained by the DEGseq methods (Supplementary Fig. S5, Supplementary Fig. S6, Supplementary Fig. S7, and Supplementary Table S13) revealed several interesting pathways, including “TGF-beta signaling” (ko04350), “insulin signaling” (ko04910), “PPAR signaling” (ko03320), “glycolysis/gluconeogenesis” (ko00010), “pyruvate metabolism” (ko00620), “peroxisome” (ko04146) and “fatty acid metabolism” (ko00071), that may form a potential regulatory network involved in determination of meat color. Therefore, particular attention should be paid to the DEGs in these pathways.

### DEGs involved in meat color formation

To date, more than 500 QTLs in the Pig QTL Database have been shown to affect meat color traits, and the number continues to increase. To further identify candidate genes associated with meat color, we performed an integrated analysis of the DEGs and the information in the Pig QTL Database. In total, 114 non-redundant DEGs were mapped to the meat color QTLs (Supplementary Table S14). Transferrin is critical in controlling the level of free iron in biological fluids, and iron is essential for heme synthesis; the transferring receptor (TfR) is a carrier protein for transferrin. Accordingly, the DEG *TfR* (ENSSSCG00000011848) may be an important gene influencing meat color. The anaerobic glycolysis reaction of glycogen takes place if the muscles are no longer supplied with oxygen, and the closely related indexes glycolytic potential and free glucose content can influence meat color^24^. Notably, *glycogen synthase 2* (*GYS2*) (ENSSSCG00000000577), a limiting enzyme in glycogen synthesis, was previously reported to be expressed only in liver and adipose tissue^27^; however, we detected it in all six skeletal muscle libraries, and it was significantly differentially expressed between Bf and Sol muscles. The function and regulatory mechanisms of *GYS2* related to meat color remain to be elucidated. Moreover, DEGs involved in fat metabolism, including *omega-3 fatty acid receptor 1* (*O3FAR1*) (ENSSSCG00000010478), *leptin* (*LEP*) (ENSSSCG00000016588) and *ELOVL fatty acid elongase 6* (ENSSSCG00000025541), should be examined closely, because lipid oxidation can facilitate myoglobin oxidation, thereby enhancing meat discoloration^28^. Additionally, the *glutathione peroxidase 2* gene (gastrointestinal) (*GPX2*) (ENSSSCG00000002279), a member of the glutathione peroxidase family, that plays a major role in protecting the organism from oxidative damage. Notably, the log 2 (fold_change) of *GPX2* between Bf and Sol was −3.5 in the present study. Several previous studies showed that antioxidants can efficiently protect lipid and myoglobin from oxidation *in vitro* and *vivo* (e.g., α-tocopherol)^28^, suggesting that the endogenous antioxidant factor GPX2 may play a critical role in the formation of meat color.

In conclusion, our data provide a comprehensive overview of the transcriptome of Bf and Sol muscles, which exhibit obvious meat color differences, and identify several potential metabolic pathways and many interesting candidate genes potentially involved in meat color traits. Overall, our results lay a solid foundation for elucidating the mechanisms of meat color formation, and the DEGs identified represent the potential valuable candidate genes for meat color traits.

## Materials and Methods

### Animal sources, tissues collection, and phenotypic characterization

The experimental population consisted of 48 Duroc × Meishan pigs derived from crossing offspring of a Duroc boar with eight Meishan sows. All pigs lived under the same condition and were raised under a standardized feeding regimen with free access to water. All procedures involving animals were carried out according to the Guide for the Care and Use of Laboratory Animals formulated by the Institutional Animal Care and Use Committee of Nanjing Agricultural University, Nanjing, China. All experiments were approved by the Institutional Animal Care and Use Committee of Nanjing Agricultural University. Main growth and carcass traits were recorded. To improve reliability and reduce inter-individual differences, three full-sib female pigs with similar growth and carcass traits were selected.

To identify candidate genes affecting meat color traits, Bf (samples Bf28, Bf35, and Bf36) and Sol (samples Sol28, Sol35, and Sol36) muscles, which exhibit obvious meat color differences, were dissected and snap frozen in liquid nitrogen prior to use. Furthermore, phenotypic differences were confirmed by quantitating the expression of *myoglobin*, and *myosin heavy chain (MyHC*) isoforms *MyHC IIb* and *MyHC I*. Sequences of quantitative primers are provided in Supplementary Table S2.

### Library construction and sequencing

Total RNA was extracted using the Trizol reagent (Invitrogen, Life Technologies, CA, USA) from Bf and Sol of three female Duroc × Meishan pigs. The RNA samples were quantitated and subjected to quality inspection. Briefly, RNA degradation and contamination were monitored on 1% agarose gels, and RNA purity and concentration were measured using a NanoPhotometer^®^ spectrophotometer (IMPLEN, CA, USA) and a Qubit^®^ 2.0 Fluorometer (Life Technologies, CA, USA), respectively; RNA integrity was assessed on an Agilent Bioanalyzer 2100 (Agilent Technologies, CA, USA).

In total, 1.5 μg of RNA for each sample was used to construct sequencing libraries, which were generated using NEBNext^®^ Ultra ^TM^ RNA Library Prep Kit for Illumina^®^ (NEB, USA) according to the manufacturer’s recommendations, and then subjected to quality assessment on an Agilent Bioanalyzer 2100. Each muscle from each of the three experimental pigs was used to prepare a sequencing library; thus six sequencing libraries were generated in total. The libraries were sequenced on an Illumina HiSeq 4000 platform after clustering and 150 bp paired-end reads were generated.

### Raw data processing and alignment analysis

High-quality clean reads were obtained by trimming the adapter sequences and removing the invalid reads containing poly-N and low-quality reads from the raw data. Meanwhile, the Q20 and GC content were calculated. All downstream analyses were conducted based on the high-quality clean reads. The clean reads were mapped to the *Sus scrofa* genome (*Sus scrofa* 10.2, http://asia.ensembl.org/Sus_scrofa/Info/Index) using HISAT (0.1.6)^29^ with default parameters. Transcripts were reconstructed using the StringTie software (1.0.4)^30^; novel transcripts were predicted by comparing reconstructed transcripts with known transcripts using the Cufflinks software (2.1.1)^31^; and coding analysis of novel transcripts was performed using the CNCI software^32^. Gene structure refinement was conducted by comparing known transcripts with reconstructed transcripts from the six transcriptome sequencing datasets using the BLAST software. AS events were detected using the rMATS software^23^.

### Identification of DEGs

First, the expression level of each gene was calculated using HTseq software (0.6.1)^33^, and normalized using the reads per RPKM method^34^. Subsequently, DEGs were identified using the R packages DEseq2 (1.4.5)^35^ and DEGseq (1.18.0)^36^, which are designed for use with biological and non-biological replicates, respectively. Supplementary Fig. S1 shows the screening strategy for DEGs. The corrected *P*-value (*Q-*value), False Discovery Rate (FDR), was used to screen the DEGs; “FDR ≤ 0.01 and absolute value of log_2_ (fold_change) ≥1” was set as the threshold to judge significance of differential gene expression. In addition, overlapping DEGs obtained from both methods were identified.

### Confirmation of DEGs

To validate the transcriptome sequencing data, a subset of DEGs were confirmed by real-time PCR. For this purpose, RNA derived from the same muscle samples used for transcriptome sequencing were subjected to quantitative analysis of gene expression. In total, 12 DEGs were randomly selected for expression analysis. Gene-specific primers (Supplementary Table S2) were designed using Primer 3 (http://bioinfo.ut.ee/primer3/). Real-time PCR assays were conducted using AceQ^®^ qPCR SYBR^®^ Green Master Mix (Vazyme, China) on a Step-One Plus Real-time PCR System (Applied Biosystems, CA, USA). All reactions were performed in triplicate and the reference gene *GAPDH* was used to normalize gene expression levels. Relative gene expression levels were calculated using the comparative Ct (∆∆Ct) value method^37^. All statistical analyses were performed using SPSS v20.0. The unpaired Student’s *t*-test was used to evaluate the statistical significance of differences between the two groups and correlation between the fold_change values of DEGs from transcriptome sequencing and real-time PCR was estimated with Pearson correlation coefficient; *P* < 0.05 was considered statistically significant difference. All data are presented as means ± standard error (SE).

### Cluster analysis and functional annotation of DEGs

Cluster analysis was conducted using the Pheatmap software in R package. Gene Ontology (GO) enrichment analysis of DEGs obtained from DEseq2 and DEGseq methods were conducted using GOseq software^38^, respectively. Meanwhile, pathway enrichment analysis was performed using the KEGG database (http://www.genome.jp/kegg/), and the hypergeometric test was used to identify significantly enriched pathways.

### QTL location analysis of DEGs

We performed QTL mapping of DEGs by comparative analysis of DEGs and porcine QTL chromosome positions using BEDTools^39^. Furthermore, the DEGs mapping to meat color QTLs were refined.

## Additional Information

**How to cite this article**: Li, B. *et al*. Identification of candidate genes associated with porcine meat color traits by genome-wide transcriptome analysis. *Sci. Rep.* **6**, 35224; doi: 10.1038/srep35224 (2016).

## Supplementary Material

Supplementary Figures

Dataset 1

Dataset 2

Dataset 3

Dataset 4

Dataset 5

Dataset 6

Dataset 7

Dataset 8

Dataset 9

Dataset 10

Dataset 11

Dataset 12

Dataset 13

Dataset 14

## Figures and Tables

**Table 1:** Output statistics and annotation information of sequencing reads.

Samples	Bf28	Sol28	Bf35	Sol35	Bf36	Sol36
Total Raw Reads	49555138	58692584	61287470	45746116	52937176	45598708
Total Raw Bases	7433234451	8803827601	9193073579	6861868795	7940534454	6839770097
Total Clean Reads	49352408	58461242	61075694	45576854	52746750	45420670
Total Clean Reads Ratio (%)	99.59	99.61	99.65	99.63	99.64	99.61
Total Clean Bases	7402826198	8769127311	9161309457	6836480369	7911972812	6813066445
Total Clean Bases Ratio (%)	99.59	99.61	99.65	99.63	99.64	99.61
Total Adatper Reads	200014	228096	208330	166640	187446	175274
Total Adatper Reads Ratio (%)	0.4	0.39	0.34	0.36	0.35	0.38
Total Low Quality Reads	2716	3246	3446	2622	2980	2764
Total Low Quality Reads Ratio (%)	0.01	0.01	0.01	0.01	0.01	0.01
Total mapped reads	35658792	43186685	44312452	33351173	38652816	33154664
Total_mapped_reads ratio (%)	72.25	73.87	72.55	73.18	73.28	72.99
Unique_mapped_reads	32771959	39616300	40857286	30702573	35553024	30481376
Unique_mapped_reads ratio (%)	66.40	67.77	66.90	67.36	67.40	67.11
Multiple mapped reads	2886833	3570385	3455166	2648600	3099792	2673288
Multiple mapped reads ratio (%)	5.85	6.11	5.66	5.81	5.88	5.89
Splice mapped reads	18590717	21283468	22403682	16390281	19845875	16751780
Splice mapped reads ratio (%)	37.67	36.41	36.68	35.96	37.62	36.88
Reads mapped in paired	31103596	37330898	37871612	28756792	33425164	28855248
Reads_mapped_in_paired ratio (%)	63.02	63.86	62.01	63.10	63.37	63.53
Read_mapped_Gene (%)	49.58	48.71	48.03	48.95	48.98	48.95
Detected_Gene_Number	16251	16785	16841	16241	16784	16402
Q20 (%)	95.78	95.38	95.16	95.14	95.30	95.70
GC content (%)	54	53.58	54.08	53.80	54.22	54.59

**Table 2:** List of 52 overlaped DEGs from DEseq2 and DEGseq methods.

GeneID	log2Fold (Bf28-vs-Sol28)	log2Fold (Bf35-vs-Sol35)	log2Fold (Bf36-vs-Sol36)	log2Fold (Bf-vs-Sol)
ENSSSCG00000025353	2.317017327	2.190707321	2.158821345	2.072881936
ENSSSCG00000006630	1.167332845	1.179529411	1.251993244	1.165069475
ENSSSCG00000006556	2.354260349	1.955909866	1.809694644	1.966469711
ENSSSCG00000006003	−1.264103162	−3.081244168	−2.090241906	−1.934687338
ENSSSCG00000000864	−1.218779653	−1.523686045	−1.081387941	−1.260454406
ENSSSCG00000002029	2.270549991	2.618479931	2.140251217	2.044228069
ENSSSCG00000008768	2.9332234	1.430576128	2.035846966	1.909964895
ENSSSCG00000006333	2.781221042	1.748589434	1.238787165	1.836938534
ENSSSCG00000025578	1.695888284	1.082628421	1.311056161	1.304621508
ENSSSCG00000011630	−1.14349131	−2.384863731	−1.974444706	−1.802578982
ENSSSCG00000028777	4.842996477	1.278759278	2.434324244	2.18033588
ENSSSCG00000022846	2.317273107	2.047028779	2.00301547	2.00300714
ENSSSCG00000017909	3.10314938	2.262848484	3.046494352	2.518839359
ENSSSCG00000009110	3.728159192	2.613375592	2.326843149	2.786016975
ENSSSCG00000001494	1.492940095	1.498975716	1.900490312	1.549775783
ENSSSCG00000025541	−1.561923854	−2.857434559	−2.159447145	−2.065202014
ENSSSCG00000000591	−1.199150794	−3.091547539	−1.899058244	−2.0165129
ENSSSCG00000000577	−2.491798139	−2.102547569	−2.134077771	−1.853100655
ENSSSCG00000003088	−1.182337451	−2.985438925	−2.375534804	−2.174141096
ENSSSCG00000016030	−3.306242388	−3.26801277	−3.275433655	−2.572357788
ENSSSCG00000023287	3.562579994	3.85438407	4.123309886	3.050462155
ENSSSCG00000015584	2.372181444	1.131917921	1.240666672	1.527150698
ENSSSCG00000029666	4.781220378	6.82191339	7.501867155	4.259422353
ENSSSCG00000022808	−1.457566576	−3.197065383	−3.441791831	−2.416185825
ENSSSCG00000013056	−1.603443249	−2.571880706	−2.658049958	−2.214436275
ENSSSCG00000026981	2.421117811	1.036177502	1.552376852	1.601215074
ENSSSCG00000022069	−1.487966071	−1.772055034	−1.570586614	−1.527993293
ENSSSCG00000027404	−1.478166364	−2.632528542	−1.595135246	−1.829280425
ENSSSCG00000024681	3.090362629	3.945149719	3.17282989	3.027690802
ENSSSCG00000009529	3.597136969	6.544699375	3.412816376	2.71743714
ENSSSCG00000016698	1.237285714	1.716880269	1.861502593	1.538379969
ENSSSCG00000027344	1.901621241	1.919907706	1.696684569	1.708438492
ENSSSCG00000015239	8.285046087	5.252933073	6.724213916	4.767166358
ENSSSCG00000010479	−1.015962426	−2.709843919	−1.936532454	−1.873350002
ENSSSCG00000023181	3.304782725	2.289770109	2.090888731	2.324073316
ENSSSCG00000011441	2.512890743	2.59048894	2.307745816	2.253737197
ENSSSCG00000005278	1.562716692	1.161298996	1.085936487	1.238186863
ENSSSCG00000024061	2.663374646	2.526170757	2.491244683	2.380976182
ENSSSCG00000027519	3.438591538	2.215402752	2.457427717	2.609781387
ENSSSCG00000002279	−2.867872248	−3.41240277	−5.515384438	−3.540000446
ENSSSCG00000010991	−2.028058787	−1.537660661	−1.277845123	−1.494322346
ENSSSCG00000026054	−1.218779284	−2.552580148	−2.084749889	−1.890689062
ENSSSCG00000015025	1.270424819	1.404894094	1.151552781	1.196180917
ENSSSCG00000009830	2.072028866	2.714597098	2.332148733	2.159353523
ENSSSCG00000028808	3.400429158	3.266473996	2.418857409	2.918846688
ENSSSCG00000030217	1.505010894	1.416013927	1.33840966	1.339181911
ENSSSCG00000024428	6.310000188	3.181548424	4.023774146	2.871379984
ENSSSCG00000004248	2.789096787	2.050189841	1.969687172	2.216113992
ENSSSCG00000013401	1.704439181	2.211645006	1.252639188	1.54508038
ENSSSCG00000000997	2.157282054	1.106447653	1.553860854	1.574494289
ENSSSCG00000010925	1.290415349	1.640401389	1.083593794	1.268643037
ENSSSCG00000011325	2.536968238	2.471171582	2.342596441	2.259156583
