# Supplementary material for: Identification of candidate genes associated with porcine meat color traits by genome-wide transcriptome analysis
Source: Sci Rep. 2016 Oct 17;6:35224. doi: 10.1038/srep35224 (PMC5066258; doi:10.1038/srep35224)
Supplement: Supplementary Figures [file srep35224-s1.pdf]

Identification of candidate genes associated with porcine meat color traits by genome-wide transcriptome analysis

Bojiang Li[1, +], Chao Dong[1, +], Pinghua Li[1], Zhuqing Ren[2, 3], Han Wang[1], Fengxiang Yu[1], Caibo Ning[1], Kaiqing Liu[1], Wei Wei[1], Ruihua Huang[1], Jie Chen[1], Wangjun Wu[1,*] & Honglin Liu[1, *]

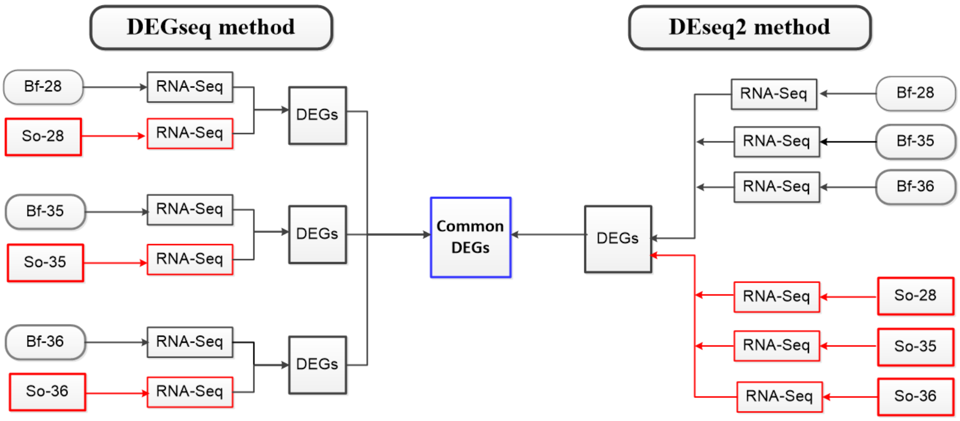

Supplementary Figure S1. Strategy of DEGs screening. Left and right parts show the workflow identified differentially expressed genes (DEGs) by DEGseq and DEseq2 methods, respectively.

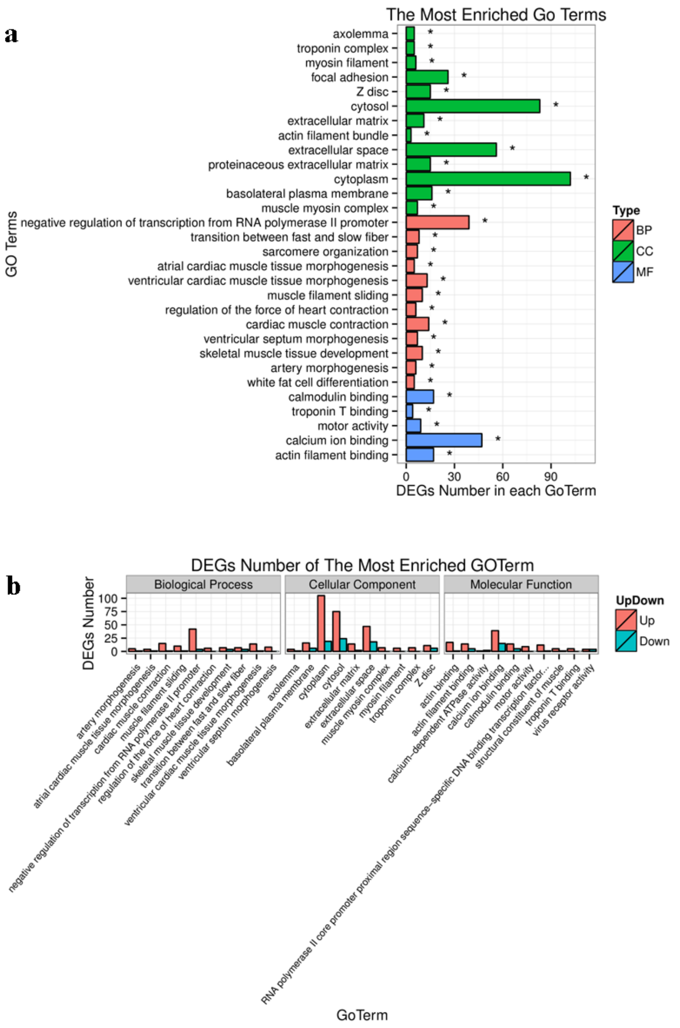

Supplementary Figure S2. Enriched GO terms and corresponding DEGs numbers of each term for Bf28-vs-Sol28. (a), The most enriched top 30 Go terms, BP: biological process; CC: cellular component; MF: molecular function. **(b),** DEGs number of the most enriched 10 GO terms derived from BP, CC and MF, respectively.

a

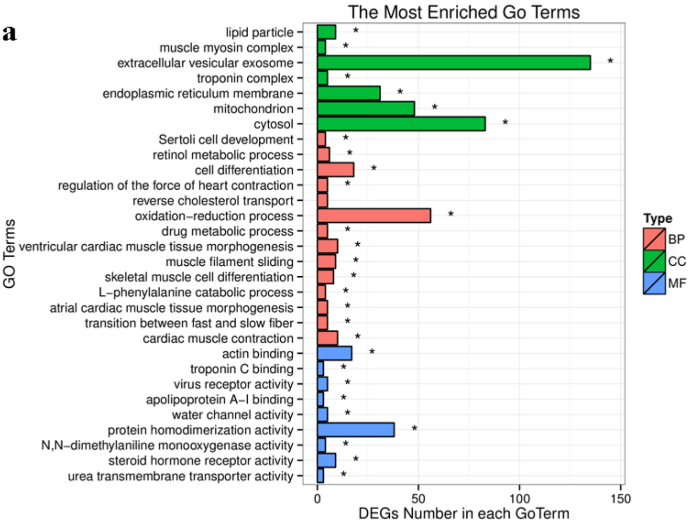

b

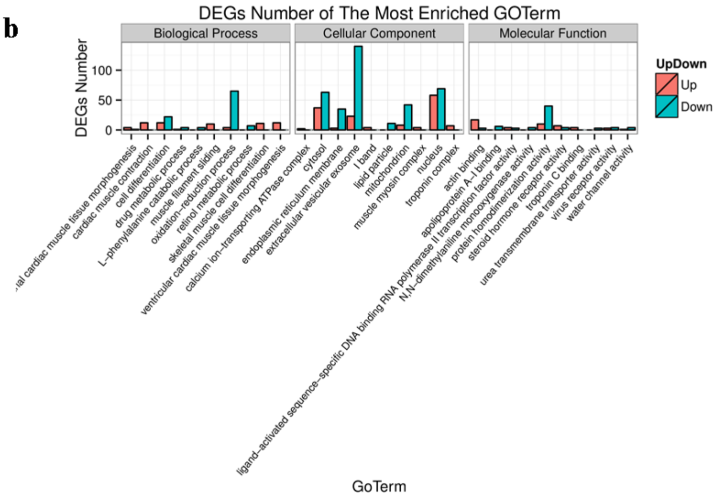

Supplementary Figure S3. Enriched GO terms and corresponding DEGs numbers of each term for Bf35-vs-Sol35. (a), The most enriched top 30 Go terms, BP: biological process; CC: cellular component; MF: molecular function. **(b),** DEGs number of the most enriched 10 GO terms derived from BP, CC and MF, respectively.

a

b

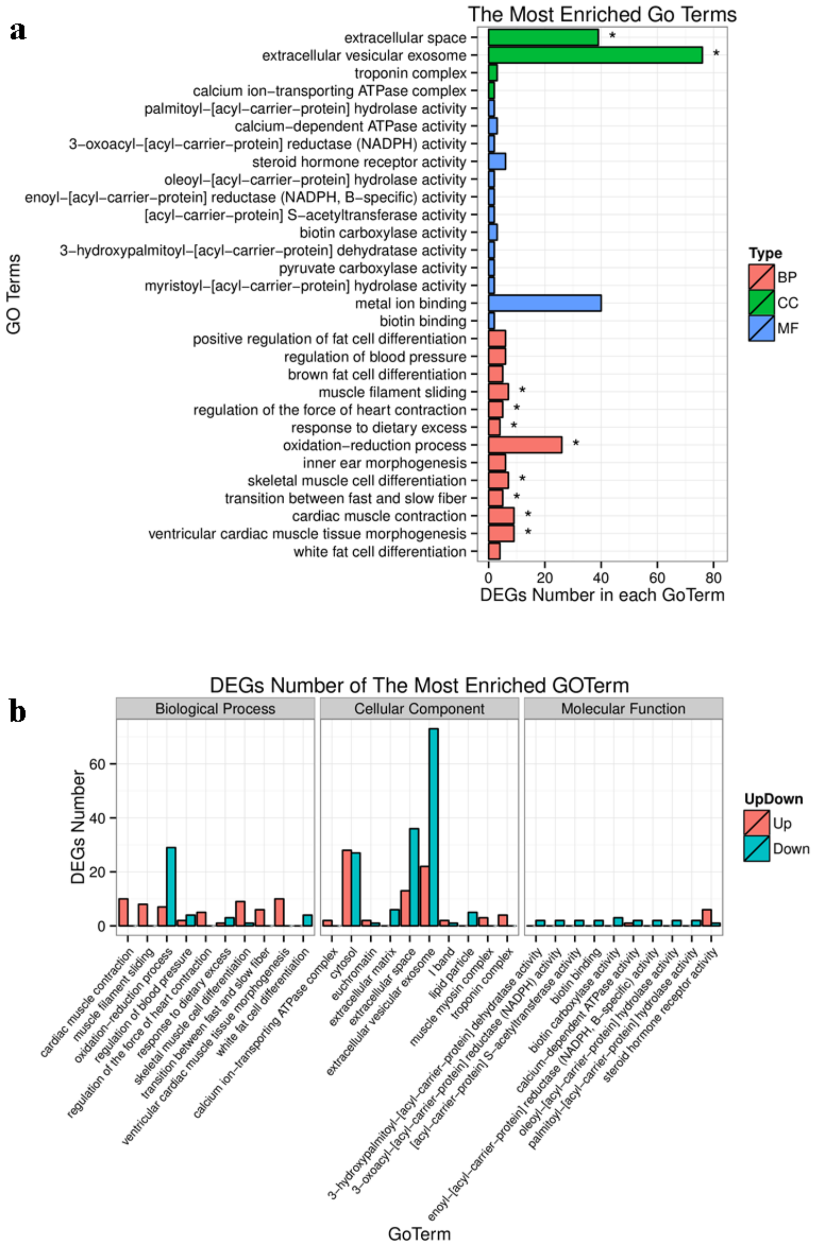

Supplementary Figure S4. Enriched GO terms and corresponding DEGs numbers of each term for Bf36-vs-Sol36. (a), The most enriched top 30 Go terms, BP: biological process; CC: cellular component; MF: molecular function. **(b),** DEGs number of the most enriched 10 GO terms derived from BP, CC and MF, respectively.

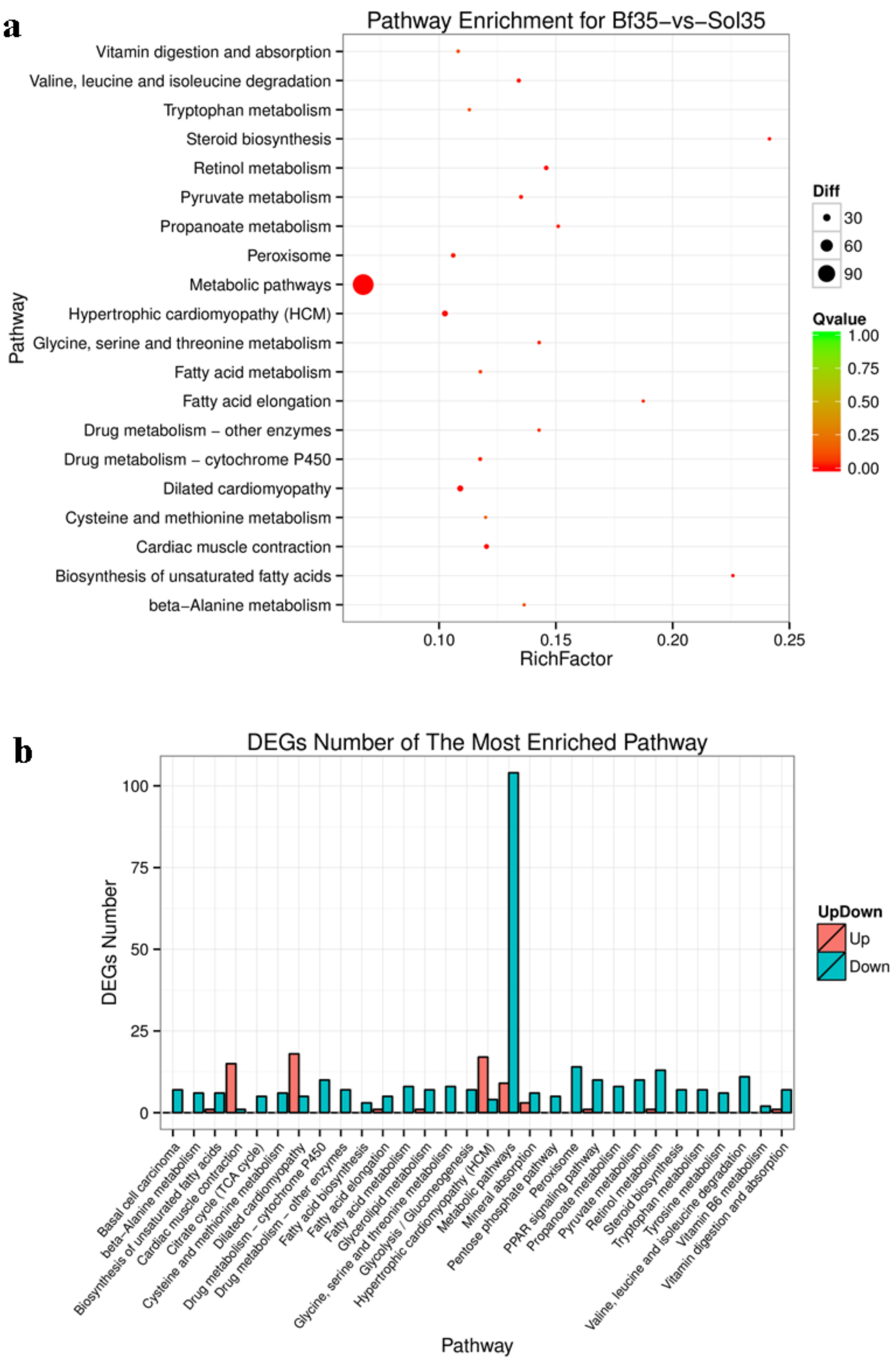

Supplementary Figure S5. Enriched pathways and corresponding DEGs number of each pathway for Bf28-vs-Sol28. (a), The most enriched 20 pathways, the size of black circle represents the DEGs number; Different color represents the significant test Q value. **(b),** DEGs number of the most enriched 30 pathways.

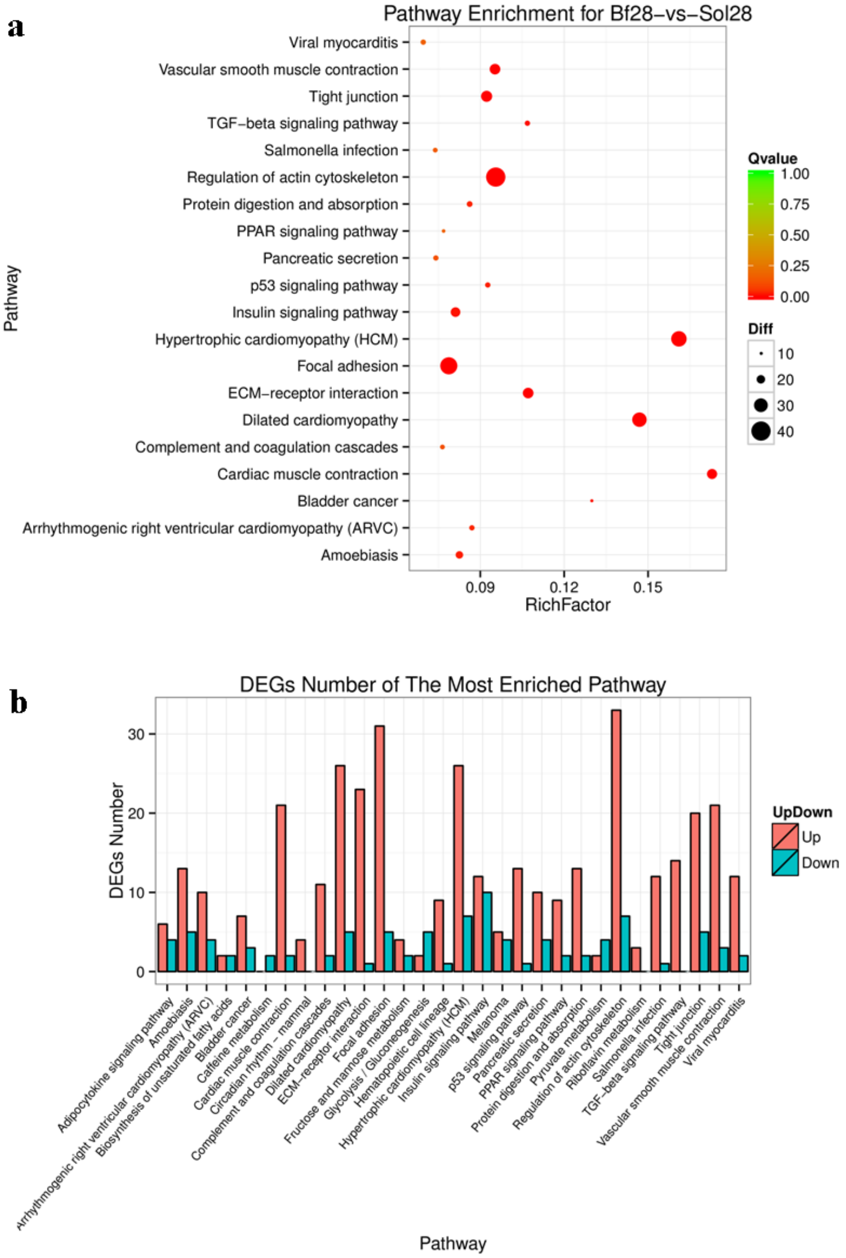

Supplementary Figure S6. Enriched pathways and corresponding DEGs number of each pathway for Bf35-vs-Sol35. (a), The most enriched 20 pathways, the size of black circle represents the DEGs number; Different color represents the significant test Q value. **(b),** DEGs number of the most enriched 30 pathways.

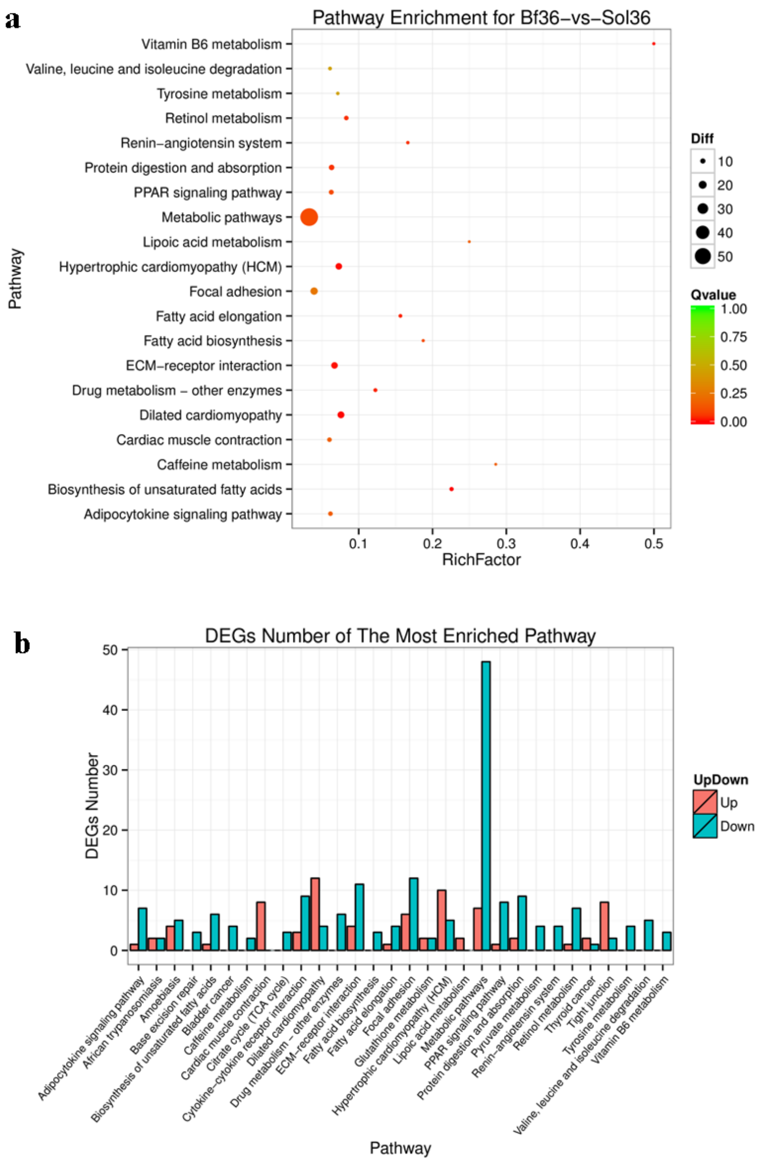

Supplementary Figure S7. Enriched pathways and corresponding DEGs number of each pathway for Bf36-vs-Sol36. (a), The most enriched 20 pathways, the size of black circle represents the DEGs number; Different color represents the significant test Q value. **(b),** DEGs number of the most enriched 30 pathways.
