# Supplementary material for: Identification of candidate genes associated with porcine meat color traits by genome-wide transcriptome analysis
Source: Sci Rep. 2016 Oct 17;6:35224. doi: 10.1038/srep35224 (PMC5066258; doi:10.1038/srep35224)
Supplement: Dataset 1 [file srep35224-s2.doc]

**Table S1. Performance of experimented pigs used in transcriptome sequencing.**

| Samples number | Sex | ADG(g/d) | BW(kg) | BL(cm) | BH(cm) | CW(kg) |
| --- | --- | --- | --- | --- | --- | --- |
| 28 | ♀ | 608.33 | 98.5 | 122.0 | 60.0 | 71.70 |
| 35 | ♀ | 629.17 | 99.0 | 112.0 | 61.0 | 74.45 |
| 36 | ♀ | 629.17 | 98.0 | 116.0 | 59.0 | 72.75 |

ADG: Average daily gain over the assessed feeding period; BW: Live weight before slaughter; BL: Body length; BH: Body high; CW: Carcass weight
