# Supplementary material for: Identification of candidate genes associated with porcine meat color traits by genome-wide transcriptome analysis
Source: Sci Rep. 2016 Oct 17;6:35224. doi: 10.1038/srep35224 (PMC5066258; doi:10.1038/srep35224)
Supplement: Dataset 2 [file srep35224-s3.doc]

**Table S2. Primers used in this study.**

| Gene name (ENSEMBL ID) | Primer sequences | Product size | Annealing temperature |
| --- | --- | --- | --- |
| *MyHC I* | F: CGACACACCTGTTGAGAAG  R: AGATGCGGATGCCCTCCA | 233 bp | 60°C |
| *MyHC IIb* | F: GTTCTGAAGAGGGTGGTAC  R: AGATGCGGATGCCCTCCA | 234 bp | 60°C |
| *Myoglobin* | F: GGATGAGATGAAGGCCTCTG  R: CCTCTCATGAACAAAGCAAC | 437 bp | 60°C |
| *TECRL*  (ENSSSCG00000024681) | F: AGATTACGCCACCCAGTG  R: TAGGCAATCCAAGAAGTGAA | 170 bp | 60°C |
| *TNNT1*  (ENSSSCG00000025353) | F: CCAGACAGGGCGTGAGATGAA  R: GAACTTCTGGGCGTGGCTGAT | 220 bp | 60°C |
| *TNNC1*  (ENSSSCG00000011441) | F: GACGAGGTGGATGAAGACG  R: TGCGGAAGAGGTCAGAAAG | 124 bp | 60°C |
| *MYOZ2*  (ENSSSCG00000009110) | F: AGGATATTCTGGACCACTG  R: CTCCTGTAATCAGGCAGTT | 177 bp | 60°C |
| *TNNI1*  (ENSSSCG00000024061) | F: ACTCCTGCTGAAGAGCCTGAT  R: CCTTGATCTCCCTGGTGTTGT | 242 bp | 60°C |
| *MYL3*  (ENSSSCG00000011325) | F: GGAAGCCAAAGCAGGAAGA  R: TCCTCATAGGTGCCCGTGT | 106 bp | 60°C |
| *CES1*  (ENSSSCG00000002825) | F: CTAACATCCCTGAGGAACTGA  R: GGGCGATACTGAAACTCATAC | 193 bp | 60°C |
| *APOE*  (ENSSSCG00000003088) | F: AGATTGCACTTGGTCAGACAGGGAC  R: GAGGGTTCTGTGGGTTG | 196 bp | 60°C |
| *ELOVL6*  (ENSSSCG00000025541) | F: CTGTGCTCCTCTACTCCTG  R: AGATGTTCGGAAAGTGAGA | 257 bp | 60°C |
| *AOX1*  (ENSSSCG00000029606) | F: GCTCCGACTCACAGGAACTAA  R: CAGCACCATACAGGGAACAGA | 152 bp | 60°C |
| *PGD*  (ENSSSCG00000003402) | F: CTGTGACTGGGTGGGAGACGA  R: ATCCTGGAACTTGAGGATGTTGG | 223 bp | 60°C |
| *Calcium-transporting ATPase*  (ENSSSCG00000023084) | F: GCCCGACTGGGACAACGAGAA  R: TGCGGACTGTGATGCCTGCT | 134 bp | 60°C |
| *GAPDH*  (NM_001206359) | F: ATCACTGCCACCCAGAAGACT  R: CATGCCAGTGAGCTTCCCGTT | 153 bp | 60°C |
